# Collagenase mRNA Overexpression and Decreased Extracellular Matrix Components Are Early Events in the Pathogenesis of Emphysema

**DOI:** 10.1371/journal.pone.0129590

**Published:** 2015-06-08

**Authors:** Fabíola S. Z. Robertoni, Clarice R. Olivo, Juliana D. Lourenço, Natália G. Gonçalves, Ana Paula P. Velosa, Chin J. Lin, Cláudia M. Fló, Beatriz M. Saraiva-Romanholo, Sérgio D. Sasaki, Milton A. Martins, Walcy R. Teodoro, Fernanda Degobbi T. Q. S. Lopes

**Affiliations:** 1 Department of Medicine, University of São Paulo Medical School, São Paulo, São Paulo, Brazil; 2 Department of Phatology, University of São Paulo Medical School, São Paulo, São Paulo, Brazil; 3 University City of São Paulo, São Paulo, São Paulo, Brazil; 4 Center for Natural and Human Sciences, UFABC, Santo André, São Paulo, Brazil; Faculty of Animal Sciences and Food Engineering, University of São Paulo, Pirassununga, SP, Brazil, BRAZIL

## Abstract

To describe the progression of parenchymal remodeling and metalloproteinases gene expression in earlier stages of emphysema, mice received porcine pancreatic elastase (PPE) instillation and Control groups received saline solution. After PPE instillation (1, 3, 6 hours, 3 and 21 days) we measured the mean linear intercept, the volume proportion of types I and III collagen, elastin, fibrillin and the MMP-1, -8, -12 and -13 gene expression. We observed an initial decrease in type I (at the 3rd day) and type III collagen (from the 6th hour until the 3rd day), in posterior time points in which we detected increased gene expression for MMP-8 and -13 in PPE groups. After 21 days, the type III collagen fibers increased and the type I collagen values returned to similar values compared to control groups. The MMP-12 gene expression was increased in earlier times (3 and 6 hours) to which we detected a reduced proportion of elastin (3 days) in PPE groups, reinforcing the already established importance of MMP-12 in the breakdown of ECM. Such findings will be useful to better elucidate the alterations in ECM components and the importance of not only metalloelastase but also collagenases in earlier emphysema stages, providing new clues to novel therapeutic targets.

## Introduction

The imbalance between proteinases and anti-proteinases is still accepted as the primary mediator of parenchymal destruction in emphysema [1,2,3,4,5] and is attested by studies in which the matrix metalloproteinases (MMPs) have demonstrated an important role in attacking the protein components of the lung parenchyma extracellular matrix (ECM) [6,7].

Even though the majority of animal models of emphysema have reinforced the importance of MMP-12 [7,8,9,10], there are many studies in patients who implicate the importance of collagenases, such as MMP-1, -8 and -13 in this disease [9,11,12].

In response to fiber destruction by MMPs, there is a structural reorganization of extracellular matrix components (ECM) in the lung parenchyma, which constitutes a dynamic process of repair and remodeling [13]. Changes in major lung ECM components, such as types I and III collagen and elastin could interfere with the mechanical properties of the lung [14]; it is believed that these changes are involved in the loss of elasticity during emphysema progression [15,16,17,18].

Although emphysema is defined by the destruction of distal air spaces, with or without fibrosis [19], the majority of clinical and experimental studies have described an increase in the amount of ECM fiber deposition in the lung parenchyma [20,21,22,23,24,25]. However, it is important to emphasize that these measurements are typically performed in patients who are already in the advanced stages of the disease and, in experimental models, only after a few days of disease induction [11,26,27,28,29].

To verify the dynamic alterations in some ECM components during emphysema development, we evaluated the volume proportion of types I and III collagen fibers, elastin and fibrillin at different time points following emphysema induction. Furthermore, we evaluated the gene expression not only for metalloelastase 12 but also for collagenases MMP-1, -8 and 13 to better understand the role of collagenases in emphysema development and progression, which remain understudied.

## Methods

This study was approved by the Ethics in Research Committee for human and animal studies of University of São Paulo School of Medicine (São Paulo, Brazil). Six- to eight-week-old male C57BL/6 mice (20–25 g) were used in this study. All animals received human care in compliance with the Guide for the Care and Use of Laboratory Animals published by the US National Institutes of Health (NIH Publication No. 85–23, revised 1996).

### Animal preparation

To induce emphysema, the animals received a nasal instillation of 50 μL (0.667 IU) of porcine pancreatic elastase (PPE) (6.6 units/mg, E-1250, Type I, Sigma-Aldrich, St. Louis, MO) [5]. The control groups received 50 μL of 0.9% NaCl (saline solution), the PPE vehicle. Mice were randomly divided into PPE group (animals that received an instillation of PPE; n = 40) and Control (S) group (animals that received an instillation of Saline Solution; n = 40). Eight animals from each group were then sampled in 1 hour, 3 hours, 6 hours, 3 days and 21 days after PPE or saline instillation.

The PPE intranasal instillation in such dose did not promote mortality and any changes in behavior of animals during the emphysema development.

To evaluate the effects of inactive PPE, we performed a new experiment to compare the alveolar enlargement in two experimental groups: Inactive PPE (n = 6) and Saline (n = 5).

For enzymatic inactivation, a 100 μL aliquot of the enzyme PPE (elastase Type I / E-1250, Sigma Aldrich) was stored in an Eppendorf tube and kept cool on ice for subsequent determination of proteolytic activity and control of the inactivation process.

To promote the inactivation of PPE enzyme and therefore its activity, the material was subjected to heating in a dry bath incubator (Major Science) for 30 minutes at 90°C.

To evaluate the process of enzymatic inactivation, the following protocol has been adopted: 96 μL of buffer (100 mM Tris-HCl, pH 8.0, containing 0.15M NaCl and 0.1% Triton X-100), 2 μL of enzyme (non-inactivated enzyme/control or enzyme inactivated by heating) and 2 μL of fluorogenic substrate (Elastase V Calbiochem—4 mM), arranged on a plate in triplicates. The outcome monitoring was performed by fluorescence spectrophotometry (Biotek-Synergy HT) using the following parameters: Sensitivity 60; Optic exposition—Top; Wavelength 380/20 (excitation) and 460/40 (emission).

Animals received a nasal instillation of 50 μL of 0.9% NaCl containing 0.667 IU of inactive PPE (6.6 units/mg, E-1250, Type I, Sigma, St. Louis, MO, USA). The control group received 50 μL of 0.9% NaCl (saline solution), the PPE vehicle.

### Lungs Preparation

At different time points the animals were anesthetized by an intraperitoneal injection of thiopental (70 mg/kg), the abdominal wall was opened and animals were exsanguinated via the abdominal aorta. For histological analysis, the thoracic cavity was opened and the lungs were removed. Then, both lungs were fixed using 10% buffered formalin infused through the trachea at a constant pressure of 20 cm H_2_0 for 24 hours, embedded in paraffin and cut into 5 μm coronal sections. Lung tissue sections were stained with H&E for lung structure analysis [30]. For PCR analysis, lungs were carefully dissected from the mediastinum and immediately immersed in RNAlater stabilizer and RNA storage solution (Applied Biosystems Canada, Streetsville, ON, Canada) at room temperature, preserved overnight at 4°C and subsequently stored at -80°C until further analysis.

### Morphometry

For conventional morphometry, an eyepiece with a coherent system of 50 lines, 100 points and a known area attached to the microscope ocular was used. The mean linear intercept (Lm), an indicator of the mean alveolar diameter [31], was assessed in 20 non-overlapping fields of lung parenchyma per animal at 200X magnification. Lm was obtained by counting the number of times that the lines of the reticulum intercepted the alveolar walls and calculated by the following equation:

Lm=Ltotal/NI

Where L_total_ is the sum of all segments of the reticulum, performed by measuring each segment with a Zeiss ruler (Carl Zeiss Microscopy GmbH, Göttingen, Germany) under a microscope with the reticulum. NI is the average number of times that the lines intersected the walls of the alveoli. Lm values were expressed in microns (μm).

We evaluated the density of the polymorphonuclear (PMN) cells in lung parenchyma by conventional morphometry using a ocular microscope with an integrating eyepiece that contains 100 points and 50 lines (point-counting technique) [32] with a known area (at_X400 magnification, 62.500 μm^2^ area). We chose 15 random parenchymal fields in each lung; we then counted the number of cells in the area and divided by the number of points hitting the lung parenchyma. The results were expressed in cells per square micrometers [25,33,34,35].

### Elastin and Fibrillin Immunohistochemistry

The tissue sections were deparaffinized and hydrated. After blocking the endogenous peroxidase activity, an antigen retrieval step was performed with pepsin at 37°C for one hour. The following primary antibodies were used in this study: a rabbit polyclonal anti-mouse fibrillin 1 (ab21618) (1:200, Abcam, Cambridge, MA, EUA) and an anti-mouse elastin A-19 (SC-17580) (1:600, Santa Cruz Biotechnology, Santa Cruz, CA, USA). The secondary antibody used for both fibrillin and elastin was VECTASTAIN Elite ABC (Vestor Elite 6105 or PK-PK-6101, Vector Laboratories, Burlingame, CA, USA) and the chromogen used was 3,3'-diaminobenzidine (DAB, Sigma Chemical Co., St. Louis, MO, USA). All sections were then counterstained with Harris’s hematoxylin. Table 1 summarizes the antibodies used for elastin and fibrillin. As negative controls, primary antibodies were omitted from the procedure and bovine serum albumin (BSA) was used instead. The images of elastin and fibrillin were captured and sent to a computer for processing using the optical microscope (Leica DM 4000B, Leica Microsystems Wetzlar GmbH, Wetzlar, Germany) coupled to a digital color camera (Leica DFC295, Leica Microsystems (Switzerland) Ltd, Heerbrugg, Switzerland).

**Table 1:** Antibodies used in immunohistochemistry studies.

Marker	Primary Antibody	Secondary Antibody
Elastin	Goat polyclonal IgG anti-mouse elastin A-19 (SC-17580) (Santa Cruz Biotechnology, CA, USA)	VECTASTAIN Elite ABC (Vestor Elite PK-6105 or PK-6101, Vector Laboratories, Burlingame, CA, EUA)
Fibrillin	Rabbit polyclonal IgG anti-mouse fibrillin 1 (ab21618) (Abcam, Cambridge, MA, EUA)	VECTASTAIN Elite ABC (Vestor Elite PK-6105 or PK-6101, Vector Laboratories, Burlingame, CA, EUA)

### Collagen I and III Immunofluorescence

Transverse sections of mouse lungs prepared in slides that were previously treated with 3-aminopropiltriethoxysilano (Sigma Chemical Co., St. Louis, MO, USA) were immersed in hot (60°C) xylol for 20 min and then submitted to three cold xylol washings and hydrated with successive washings in ethanol at decreasing concentrations (100%-75%), distilled water and phosphate buffer (PBS). For the exposition and recovery of the antigenic sites, the material was digested with pig pepsin (10,000 U/ml) (Sigma Chemical Co., St. Louis, MO, USA) dissolved in 1 mM acetic acid for 30 min at 37°C. The treated sections were washed three times, for 10 min each with PBS and then incubated with anti-collagens I and III rabbit polyclonal antibodies (Table 2), diluted at 1:60 and 1:30 in PBS, respectively, during the night. The specificity of the antibodies was validated by Western blot [36]. After this incubation, the cuts were washed in PBS with 0.05% Tween^20^ and incubated for 60 min with anti-IgG secondary antibody (Alexa 488-conjugated goat anti-rabbit IgG, 1:200, Invitrogen, Life Technologies, Eugene, OR, USA) containing 0.006% Evans blue (Table 2). After being cover-slipped in fluorescence mounting media, the signal was detected by fluorescence microscopy using an Olympus BX51 microscope (Olympus Co, Tokyo, Japan). As a control, PBS was used in place of the primary antibody.

**Table 2:** Antibodies used in immunofluorescence studies.

Marker	Primary Antibody	Secondary Antibody
COL I	Rabbit polyclonal anti-Collagen type I (Rockland, Limerick, PA, USA)	Alexa Fluor 488 goat anti-rabbit IgG (Invitrogen, Life Technologies, Eugene, OR, USA)
COL III	Rabbit polyclonal anti-Collagen type III (Extracellular Matrix Lab, FMUSP, Sao Paulo, BR)	Alexa Fluor 488 goat anti-Rabbit IgG (Invitrogen, Life Technologies, Eugene, OR, USA)

### Image Analysis

Quantitative histological measurements were made with the image analysis software Image-Pro Plus 4.5 for Windows (Media Cybernetics, Inc., Silver Spring, MS, USA). For each ECM fiber (elastin, fibrillin, type I collagen and type III collagen), ten random and non-overlapping fields were evaluated for all mice. The total amount of each fiber type was expressed as the area of fiber divided by the total parenchyma area observed and multiplied by 100. The final result was the average of the evaluated fields for each animal.

### Metalloproteinase gene expression

For this analysis, we selected matrix metalloproteinase (MMP) target genes related to the degradation of ECM lung fibers analyzed in this study. TaqMan Gene Expression Assay (Life Technologies, Carlsbad, CA) was used to assess the expression of the following MMPs: *MMp1a* (NM_032006.3; assay Mm00473485, Life Technologies), *MMp1b* (NM_032007.3; assay Mm00473493, Life Technologies), *MMp8* (NM_008611.4; assay Mm00439509_m1, Life Technologies), *MMp12* (NM_008605.3; assay Mm00500554_m1, Life Technologies) and *MMp13* (NM_008607.1; assay Mm00439491_m1, Life Technologies).

The extraction of total RNA was performed by using a commercially available acid guanidinium thiocyanate-based single step method (TRI-Reagent MRC, Cincinnati, OH, USA) according to the protocol provided by the manufacturer. The concentration of obtained RNAs was estimated by absorbance at 260 nm in spectrophotometry and RNA integrity was confirmed by electrophoresis on a 1% native agarose gel for all experimental groups. Aliquots of 5 μg total RNA were reverse transcribed using the High Capacity RNA-to-cDNA Master Mix Kit (Applied Biosystems, Life Technologies, Grand Island, NY, USA) following the manufacturer's instructions.

Samples from each animal were assayed in triplicate and all real-time PCR was performed on the StepOnePlus Real-Time PCR System (Applied Biosystems, Foster City, CA, USA). Polymerase chain reactions for target genes were performed in 20 μL of 1X TaqMan reaction buffer (TaqMan Gene Expression Master Mix, Applied Biosystems, Foster City, CA, USA) using 2 μL of cDNA. Relative quantification of the expression of target genes was obtained by using the 2^-ΔΔC^_T_ approach [37] with GAPDH as an endogenous control gene. Unlike the target genes, which used the TaqMan Gene Expression Assay, SYBR Green (SYBR Green Master Mix, Applied Biosystems, Foster City, CA, USA) was used as a fluorescent dye to detect amplification of GAPDH mRNA.

Amplification efficiencies of both target and control genes were determined by standard curves of amplification using the Ct obtained after serial dilutions of one cDNA sample. The equivalence of target genes and GAPDH amplification efficiencies was verified by comparing the slope of the standard curves.

### Statistical analysis

A statistical analysis was performed using SigmaPlot software (SPSS Inc. Chicago, Illinois, USA). Statistical significance of differences between groups was assessed with Student’s *t*-test or the Mann–Whitney *U* test analysis. All data were presented as the means and standard deviations (Mean + SD). A value of p≤0.05 was regarded as statistically significant.

## Results

### Progressive increase of the Mean Linear Intercept (Lm)

**Fig 1:**
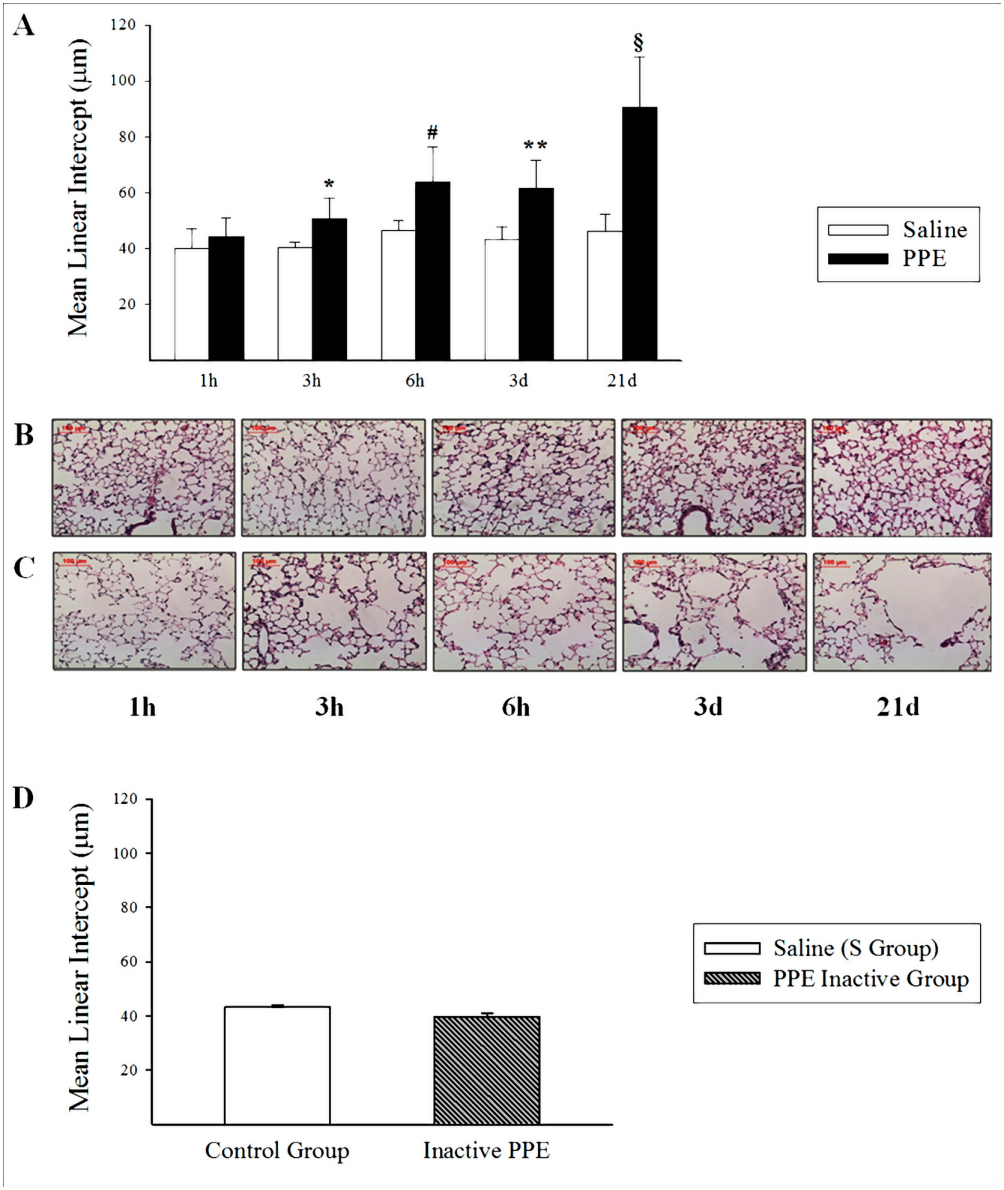
Mean linear intercept (Lm) values measured in all S and PPE groups (A) and photomicrographs of mice lung parenchyma (B and C). A) *p = 0,021; ^#^p<0.001; **p = 0.003; ^§^p<0.001; all compared to respective Control group (S). Values are means and SD. B) Photomicrographs of lung parenchyma in S groups at all protocol times. C) Photomicrographs of lung parenchyma in PPE groups at all protocol times. There was an increase in Lm in the PPE groups compared to their respective S controls. (400X magnification, hematoxylin-eosin staining). D) Mean linear intercept (Lm) values measured in Control group (S) and inactive PPE groups. Values are means and SD.

An increase in Lm values was detected in all PPE groups from the 3rd hour after PPE instillation and remained until the 21st Day. Fig 1A shows the mean linear intercept values (Lm) measured in the experimental groups (PPE-3h: *p = 0.021; PPE-6h: ^#^p<0.001; PPE-3d: **p = 0.003; PPE-21d: ^§^p<0.001) compared to the respective Control (S) groups. The emphysematous lesions showed a heterogeneous distribution within the lung parenchyma. Fig 1B and 1C show photomicrographs of lung parenchyma in all Control (S) and PPE groups, highlighting the progressive increase in the distal air spaces in PPE compared to the S controls.

The Fig 1D shows the mean linear intercept values (Lm) measured in the inactive PPE and Control (S) groups. We did not detect statistical differences comparing these experimental groups, suggesting that inactive PPE is comparable with saline solution.

### Increase in PMN in lung parenchyma

**Fig 2:**
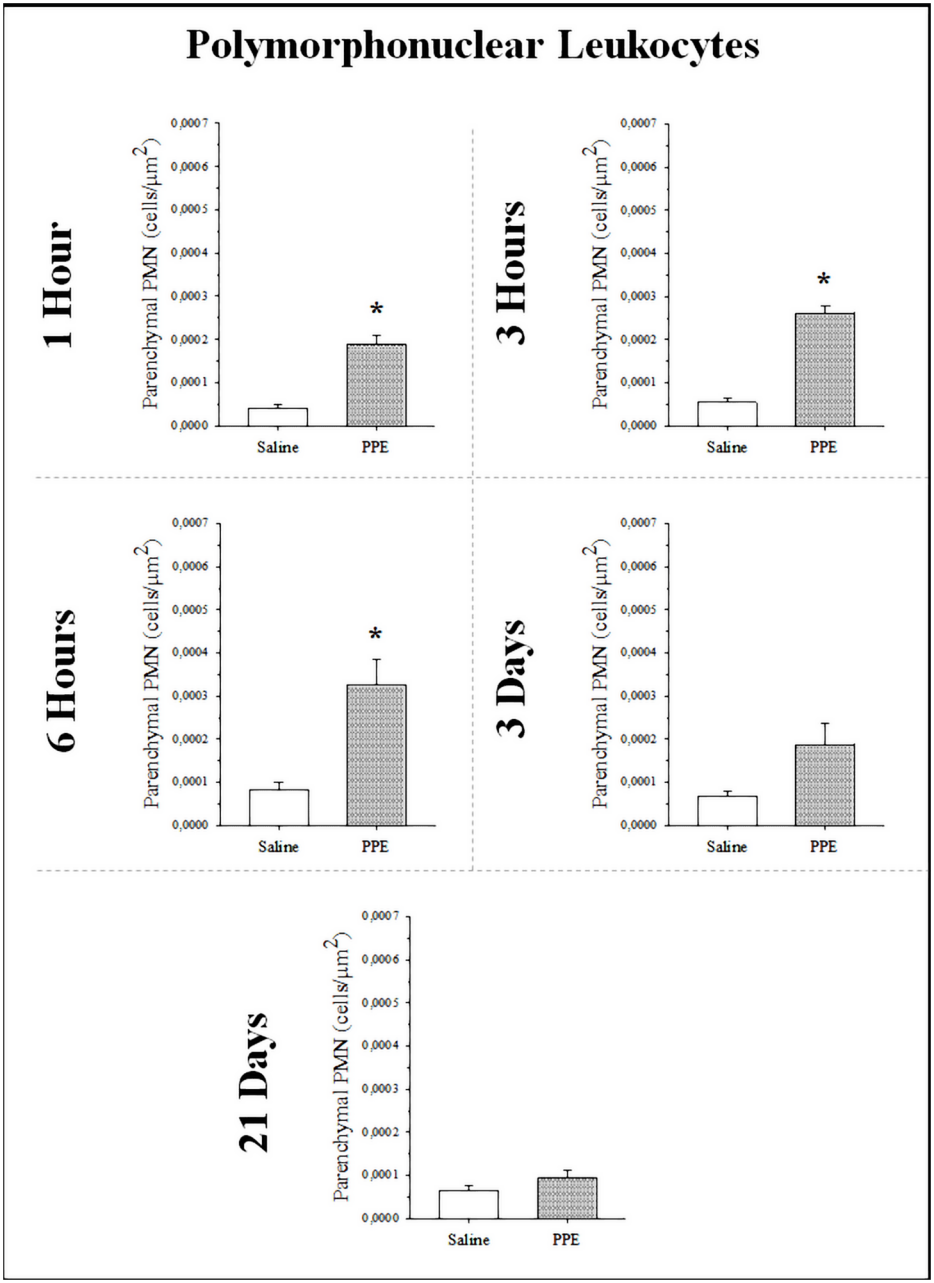
The PMN density values are shown in all protocol groups (values as the means and SD). There was an increase in PMN density in parenchyma in PPE groups compared to respective Control groups in 1h (* p< 0.001), 3 h (* p<0.001), 6 h (* p = 0.008) and 3 d (*p = 0.015).

The density of PMN in lung parenchyma was increased since the 1^st^ hour after the PPE instillation and it was remained until the 3^rd^ day (Fig 2) in groups PPE-1h (*p<0.001), PPE-3h (*p<0.001), PPE-6h (*p = 0.008), PPE-3d (*p = 0.015) compared the respective control groups S-1h, S-3h, S-6h and S-3d. There was no difference between the experimental PPE and Control (S) groups at the 21^st^ day.

### Increase in metalloproteinase gene expression

**Fig 3:**
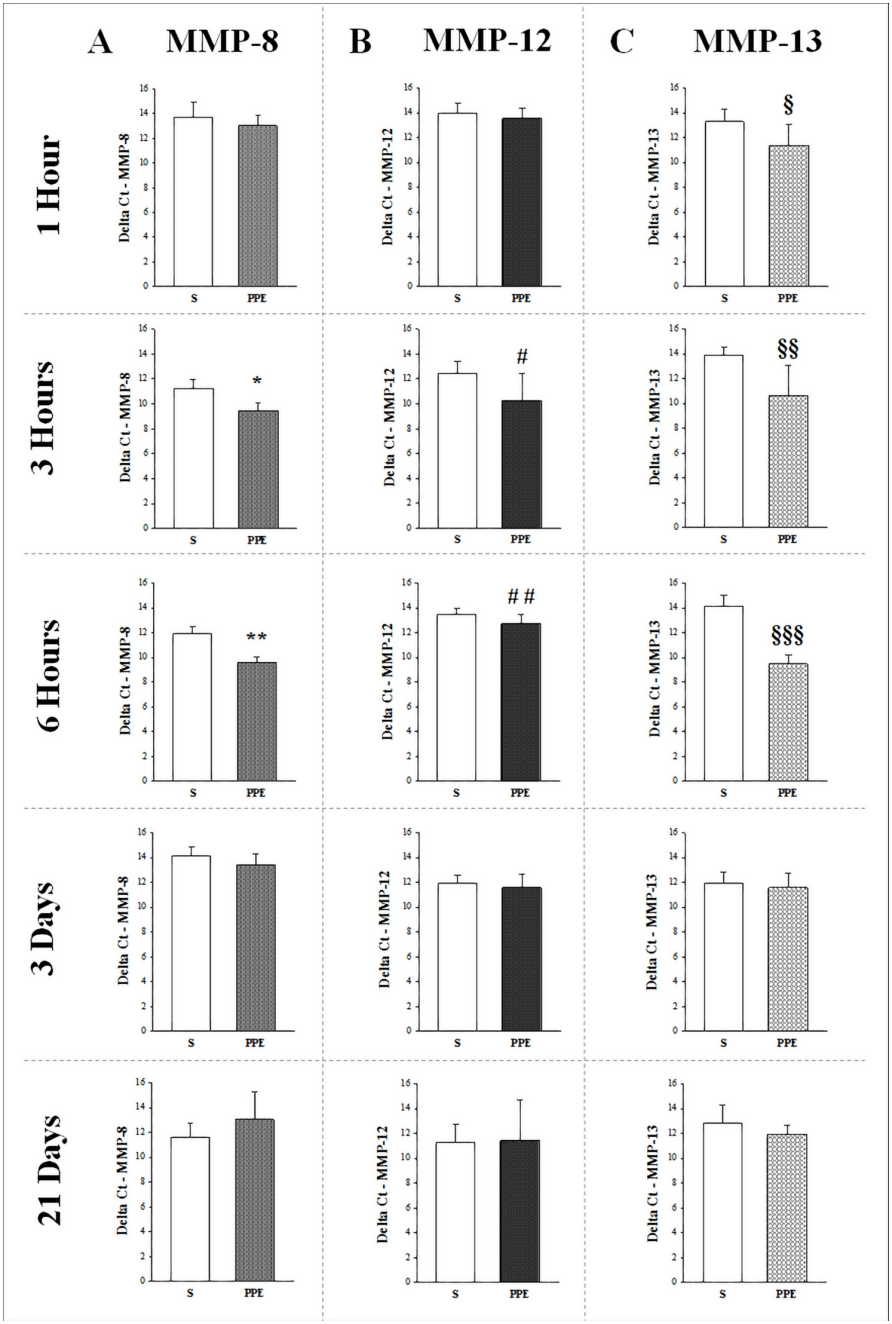
Metalloproteinase gene expression at different time points expressed as ΔCt. A) MMP8: *p<0.001 compared to respective S group and **p<0.001 compared to respective S group. B) MMP12: ^#^p = 0.033 compared to respective S group and ^##^p = 0.035 compared to respective S group. C) MMP13: ^§^p = 0.021 compared to respective S group; ^§§^p = 0.007 compared to respective S group and ^§§§^p<0.001 compared to respective S group. All values are means and SD.

The metalloproteinase gene expression analysis showed an increase in MMP-8 (Fig 3A) in groups PPE-3h (*p<0.001) and PPE-6h (**p<0.001) compared to the respective control groups S-3h and S-6h. Additionally, there was an increase in MMP-12 gene expression (Fig 3B) in groups PPE-3h (^#^p = 0.033) and PPE-6h (^##^p = 0.035) compared to the control groups. There was no statistically significant difference between the ΔCT values of the PPE and Control (S) groups at 1 hour, 3 days and 21 days for both MMP-8 (Fig 3A) and MMP-12 (Fig 3B). Delta CT analysis for MMP-13 (Fig 3C) revealed increased expression in PPE groups at 1 hour (^§^p = 0.021) and 3 hours (^§§^p = 0.007), and remained increased until 6 hours (^§§§^p<0.001) compared to their respective S control groups. There was no difference between the experimental PPE and Control (S) groups at 3 days and 21 days (Fig 3C). MMP-1 gene expression was also evaluated through its subtypes MMP-1a an MMP-1b, however, no statistically significant differences were found between the experimental groups.

### Immunohistochemical and immunofluorescence analysis—Volume proportion of collagen and elastic fibers decreases before increasing

#### Fibrillin and elastin

**Fig 4:**
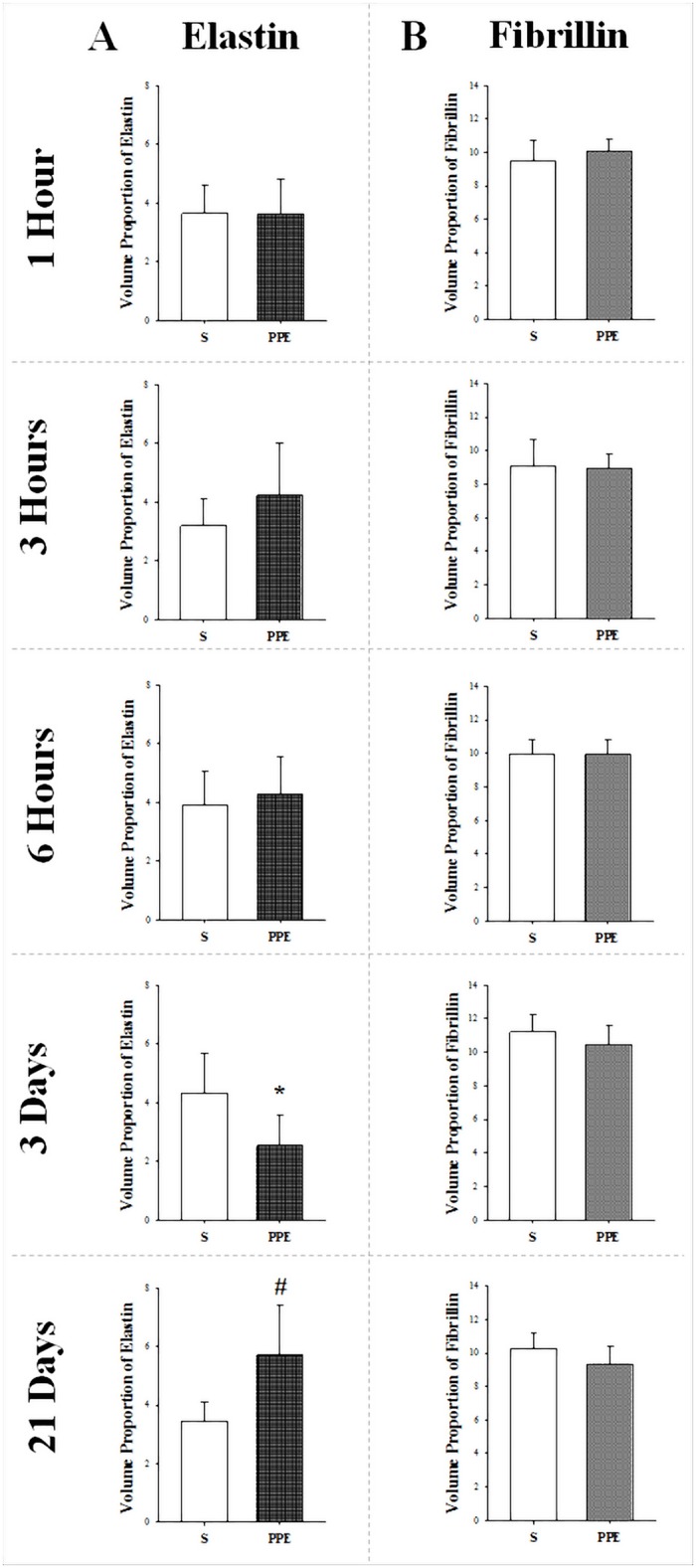
Volume proportion of elastin (A) and fibrillin (B) are shown in all protocol groups (values as the means and SD). A) Elastin: *p = 0.014 compared to respective S group and ^#^p = 0.012 compared to respective S group. B) Fibrillin: There was no significant difference in any of PPE groups compared to their respective S groups.

**Fig 5:**
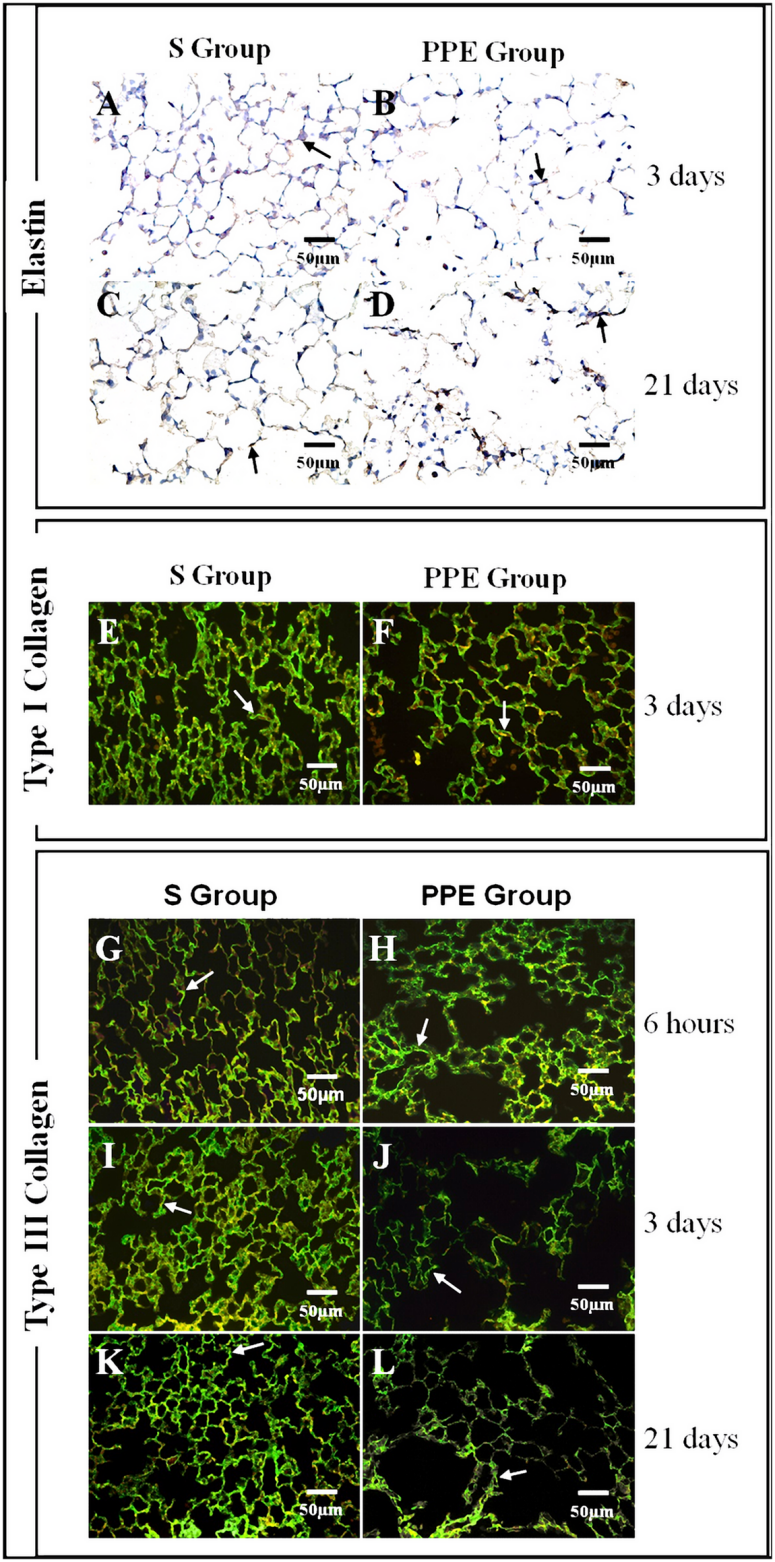
Photomicrographs of Elastin, Type I Collagen and Type III Collagen. A-D) Photomicrographs of elastin in parenchyma (H&E staining, 400X magnification). There was a decrease in elastin amount at the 3^rd^ day with a posterior increase in such fibers at 21^st^ day, comparing the PPE with S group. E-F) Photomicrographs of type I collagen staining by immunofluorescense in alveolar tissue (400X magnification). There was a decline in those fibers at the 3^rd^ day in PPE group; G-L) Photomicrographs of type III collagen staining by immunofluorescense in alveolar tissue (400X magnification). There was a decline in type III collagen at the 6^th^ hour and 3^rd^ day with a consecutive increase at the 21^st^ day in PPE compared with S groups.

The volume proportion of elastin in lung parenchyma showed a decline in group PPE-3d (*p = 0.014) compared to its respective control group S, while after 21 days an increased proportion of elastin in the lung was observed in animals that received an instillation of PPE (PPE-21d: ^#^p = 0.012) compared to those that received an instillation of saline. There was no significant difference in the proportion of elastin in the parenchyma between the PPE and Control (S) experimental groups for the other time points (Figs 4A and 5A–5D). There was no statistically significant difference in volume proportion of fibrillin in any of the PPE groups with respect to their S controls (Fig 4B).

#### Type I and Type III Collagen

**Fig 6:**
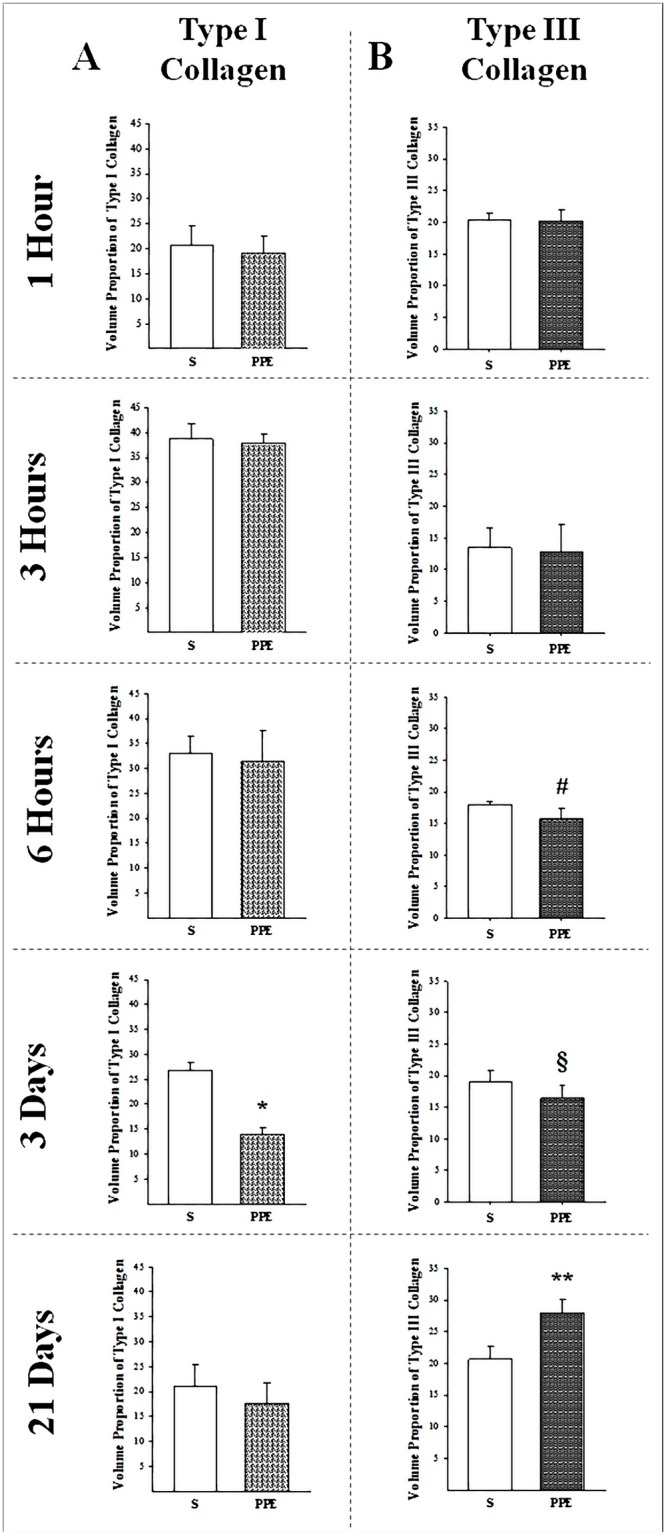
Volume proportion of type I and III collagen are shown in columns A and B for all protocol groups. A) Type I Collagen: *p<0.001 compared to respective S group. B) Type III Collagen: ^#^p = 0.002 compared to respective S group; ^§^p = 0.038 compared to respective S group; **p<0.001 compared to respective S group. Values are means and SD.

There was a decrease in the volume proportion of type I collagen in the lung parenchyma of the PPE-3d group (*p = 0.032) compared to the S-3d control group (Figs 6A, 5E and 5F). There was no statistically significant difference between other Control (S) and PPE groups. Whereas the volume proportion of type III collagen showed a decrease in PPE-6h (^#^p = 0.002) and PPE-3d (^§^p = 0.038), there was an increase in group PPE-21d (**p<0.001) with respect to their controls. There was no statistically significant difference between the Control (S) and PPE groups at 1 hour (Figs 6B and 5G–5L).

## Discussion

In the present study, our analysis revealed an initial decrease in ECM fibers amount, which had occurred at times later than when we detected increases in polimorphonuclear leukocytes in parenchyma and in gene expression for collagenases MMP-8, -13 and for metalloelastase-12; only 21 days following emphysema induction we detected increases in some of the ECM fiber amounts in PPE groups. Furthermore, the increase in distal air spaces (Lm) induced by an instillation of PPE occurred early in this experimental model, starting at 3 hours where it remained for the duration of the experiment (6 hours, 3 days and 21 days).

We observed an increase in PMN leukocytes since the first hour after the PPE instillation, suggesting an inflammatory process that was remained until the 3^rd^ day. Some studies showed the association between the PMN recruitment into the lung and the onset of alveolar walls destruction [38,39,40]. Probably it occurs due the ECM fibers attack by proteases, such as metalloproteinase-8, delivered by neutrophils [7].

We found a decrease in type III collagen from the first hours of emphysema induction (from the 6th hour until the 3rd day) and in type I collagen at the 3rd day. These decreases in collagen amount occurred at time points posterior to the observed MMP-8 and -13 gene expression increases in animals that received PPE instillation compared to controls. Only at the 21st day the type III collagen fibers showed an increase compared to the S group; the type I collagen returned to values similar to the control groups.

Such findings might help explain why in animal models of emphysema, collagen fibers break at tensions that correspond with normal breathing [5]. Type I and III collagen fibers are the major structural elements of the lung and are fundamental for maintaining normal lung architecture, which suggests that alterations in alveolar wall structure are consequences of changes in the collagenous composition of the tissue [41]. Indeed, some studies showed that the fiber stiffness depends on the relative amounts of type I and type III collagens, since type I collagen is stiffer than type III [17,42].

The increase in gene expression for MMP-13 and -8, from the first and 3^rd^ hour after PPE instillation, respectively, revealed the importance of such collagenases in the onset of parenchymal destruction. Until now, previous studies showed an up-regulation of MMP-8 and -13 in mice only after prolonged exposition to cigarette smoke [10] or in the lungs of patients with established COPD [43,44].

In addition, we found an increase in gene expression for MMP-12 at earlier times (3 and 6 hours), which corresponded with a reduced proportion of elastin in the lung (3 days) in animals that received PPE instillation, reinforcing the already established importance of MMP-12 in the breakdown of this ECM component [10,45]. On the other hand, we did not observe differences between the experimental groups at the different time points when evaluating the deposition of fibrillin. Some studies suggest that these proteins are also substrates for MMP-13 [46], and although we have demonstrated increased expression for this MMP in our experimental model, we did not observe a relationship between the increase of this metalloproteinase and the amount of fibrillin.

After 21 days of PPE instillation we observed an increase in the proportion of elastin in mice lungs, which is in agreement with previous studies [22,25]. However, many studies indicated that despite the increased elastin content after emphysema development, these fibers are non-functional due to their molecular complexity, which renders repair processes of these fibers inefficient leading to impairment of lung function [16,47], as showed previous by our group after 21 days of the emphysema induction using this same experimental model [30].

Interestingly, we did not find an up-regulation at any time point for MMP-1 in this experimental model. Since D'Armiento *et al* [48] showed development of emphysema shortly after birth in a transgenic mouse model that overexpressed human MMP-1 in the lung, this MMP is considered important in emphysema development. In another study, Shiomi [49] used a heterozygous line from a transgenic mouse model that overexpressed human MMP-1 and observed development of emphysema at 12 months with a decrease in type III collagen and increased lung compliance. They suggested that the loss of collagen III was a greater determinant of emphysema than the loss of collagen I. It is likely that we did not observe an up-regulation for MMP-1 gene expression because we used wild type animals in this experimental model.

To our knowledge, this is the first study that described a reduction in ECM fibers at earlier time points following emphysema induction but prior to the increase in their deposition, which suggests that the destruction and repair processes do not occur simultaneously in lung parenchyma. Additionally, we showed that there is an interval of time between the increases in MMP gene expression and in the increased amount of parenchymal fibers.

Considering that the parenchymal fibers did not show functional effectiveness in respiratory mechanical maintenance after the remodeling process, this study could provide new clues to elucidate at which time points during emphysema development will be possible candidates to test new therapeutic strategies, considering the importance of collagenases (MMP-8 and -13) and metalloelastase MMP-12 in emphysema development.
